# The Performance Evaluation of Asphalt Mortar and Asphalt Mixture Containing Municipal Solid Waste Incineration Fly Ash

**DOI:** 10.3390/ma15041387

**Published:** 2022-02-14

**Authors:** Xiaowen Zhao, Dongdong Ge, Jiaqing Wang, Dianwen Wu, Jun Liu

**Affiliations:** 1 Hunan Provincial Communications Planning, Survey & Design Institute Co., Ltd., Changsha 410200, China; zhaoxiaowen2013@163.com (X.Z.); dianwenwu@163.com (D.W.); 2 National Engineering Laboratory of Highway Maintenance Technology, School of Traffic & Transportation Engineering, Changsha University of Science & Technology, Changsha 410114, China; 3 College of Civil Engineering, Nanjing Forestry University, Nanjing 210037, China; jiaqingw@njfu.edu.cn; 4 Louisiana Transportation Research Center, Louisiana State University, 4101 Gourrier Ave, Baton Rouge, LA 70808, USA

**Keywords:** asphalt mortar, asphalt mixture, fly ash, high temperature performance, low temperature property, moisture susceptibility

## Abstract

The aim of the research is to quantify the property of asphalt mortar and asphalt mixture containing municipal solid waste incineration (MSWI) fly ash. The potential of partially replacing mineral fillers with MSWI fly ash in asphalt mixture production was investigated. Five different MSWI fly ash replacement ratios, which include 0%, 25%, 50%, 75%, and 100%, were adopted to assess the influence of fly ash dosage, and the optimum fly ash replacement ratio was proposed. The rheological characteristics of asphalt mortar with MSWI fly ash were assessed with the dynamic shear rheometer (DSR) and bending beam rheometer (BBR). The high temperature properties of the mixture with MSWI fly ash were assessed with the Marshall stability test and the rutting test. The low temperature cracking property was determined with the indirect tensile strength test at low temperatures. The moisture stability property was identified with the immersed Marshall test and the freeze-thaw cycles conditioned indirect tensile strength test. Based on the test results, the addition of fly ash and mineral filler remarkably increased the ǀG*ǀ of the asphalt mortar. The δ of asphalt decreased as the dosage of fly ash and mineral filler increased. The addition of fly ash and mineral filler degraded the low temperature characteristics of the mortar. Fly ash improved the high temperature characteristics of the asphalt mixture. The asphalt mixture with MSWI fly ash was more susceptible to thermal cracking than the control sample. The addition of fly ash weakened the moisture stability of the asphalt mixture. In order to guarantee the low temperature characteristics and the moisture susceptibility of the asphalt mixture, the fly ash replacement ratio was recommended to be set around 25%. With proper mixture design and fly ash dosage, the asphalt mixture would have adequate performance, as well as reduced environmental impact.

## 1. Introduction

With city expansion and increasing populations in municipal cities, the amount of new solid waste generated bring a significant issue to cities [1]. Using incineration to process solid waste was the most effective method. Handling solid waste with landfill method wasted precious land and threatened groundwater. After the incineration process, the solid waste included fly ash and bottom ash, based on the location where the ash was collected [2]. Bottom ash is the residue in the bottom of the incinerator, which consisted of slag, glass, metal, and incompletely burned organic components [3]. The fly ash is the fine particles received from the gas purification system of the incinerator, which accounts for 10–20% of the solid waste incineration residue [4]. The characteristics of the fly ash from different incinerators vary because the components of solid waste are different [5]. However, the untreated fly ash normally had low moisture content and had grey or white appearance. During the incineration procedure, the heavy metal components were concentrated in the fly ash [6]. If treated improperly, the fly ash will contaminate the environment and influence the health of human beings.

Fly ash has long been adopted as road construction material. By adopting fly ash in the asphalt mixture and concrete construction, the demanding using natural aggregate and mineral fillers could be reduced [7]. With the consumption of natural materials, the reduction of the yield of original materials increased the cost of construction. Using fly ash in pavements will reduce the cost and relieve the pressure of the fly ash on the environment. Fly ash can be applied as supplementary materials in the concrete production [8]. The main components of fly ash were similar to cement materials, which proved the commercial potential to partially replace cement in the concrete production [9]. The fly ash contributed to the strength of concrete because of the hydraulic and pozzolanic reaction [10,11]. During concrete production, fly ash can replace part of the fine aggregate, but the heavy metal stabilization efficiency was not sufficient. The heavy metal in fly ash was still easily leached out from the concrete. Due to the good bonding property of asphalt binder, asphalt binder was proved to be more effective to stabilize the heavy metal in fly ash [12].

Fly ash has been treated as filler in the asphalt mixture production [13,14]. When mixing fly ash with asphalt mixture, the fly ash interacted with the asphalt binder, and the stiffness of the asphalt mastic was improved [15,16]. The fly ash absorbed the light components and finally reduced the flowability of the asphalt binder [17]. The interface condition between asphalt and aggregate was affected by the addition of the fly ash [18]. The asphalt film thickness of the aggregate was increased and thus increased the modulus of the asphalt mixture [19]. The production property and the serviceability of the asphalt mixture were improved with the adoption of fly ash during the design and the placement of the asphalt mixture [20,21]. The stiffness was enhanced with the adoption of fly ash, and the stability of the asphalt mixture was influenced by the dosage of fly ash in the asphalt mixture [22,23]. The physical property of asphalt mixture was enhanced by using the fly ash in the asphalt mixture, and the improved efficiency was affected by the asphalt binder and fly ash resource [24,25]. The moisture damage resistance of the asphalt mixture was influenced by the mixture with fly ash design. Some research proved that fly ash could be utilized as effective anti-stripping agents to improve the moisture damage resistance of the asphalt mixture [26]. The moisture resistance of the asphalt mixture was influenced by the dosage of the fly ash and the mixture design [27].

The objective of the research is to quantify the properties of asphalt mortar and asphalt mixture containing municipal solid waste incineration (MSWI) fly ash. The rheological characteristics of asphalt mortar with MSWI fly ash were assessed with the dynamic shear rheometer (DSR) and bending beam rheometer (BBR). The high temperature property of the asphalt mixture was assessed with the Marshall stability test and the wheel tracking test. The low temperature performance of the mixture was assessed with the indirect tensile strength test.

## 2. Materials and Methods

### 2.1. Materials

The asphalt binder used in the paper was penetration grade #70. Table 1 lists the binder performance.

The coarse aggregates used were limestone from Hunan in China, and the basic performances of the coarse aggregate are presented in Table 2.

The properties of limestone mineral fillers and MSWI fly ash used in this study are shown in Table 3. The MSWI fly ash has lower apparent specific gravity than the limestone mineral filler. When mixed with asphalt binder, the MSWI fly ash was more likely distributed uniformly and had less tendency to separate from the asphalt binder than limestone mineral filler. The surface area of the MSWI fly ash was 4.66 times higher than that of limestone mineral filler. The larger surface area guaranteed strong adhesion between the MSWI fly ash and the asphalt binder.

The element component of the MSWI fly ash was measured with the AA-6800 Atomic Absorption Spectrophotometer, and the results are presented in Table 4. The MSWI fly ash had many heavy metals, which included lead (Pb), copper (Cu), cadmium (Cd), nickel (Ni), chromium (Cr), and zinc (Zn), which are harmful to the biological system and the environment. By mixing MSWI fly ash with asphalt binder, the toxic heavy metals can be effectively stabilized and solidified, which mitigated the burden of heavy metals as hazardous pollutants to the environment [28].

### 2.2. Test Methods

#### 2.2.1. Asphalt Mortar Production

In order to assess the impact of MSWI fly ash and mineral filler on the properties of asphalt binder, asphalt mortar was prepared by mixing asphalt binder with fly ash and mineral filler with three different ratios of fly ash and mineral filler to asphalt (F/A, 0.6, 0.8, and 1.0 by weight). Different fly ash and mineral filler ratios were adopted to explore the optimum fly ash dosage, Table 5 shows the material matrix.

The base asphalt was heated to 135 °C, and the fly ash and mineral filler were heated to 140 °C. Fly ash and mineral filler were added into asphalt binder based on the F/A ratios and the dosage of fly ash and mineral filler in Table 5. The speed of the high shear mixer was 1000 RPM during the materials adding process. The mortar was then mixed at 3000 RPM for 10 min. Finally, the mortar was sheared at 1000 RPM for 5 min to remove air bubbles imported during the high shear mixing process. Each test condition had three replicate samples.

#### 2.2.2. Asphalt Mortar Properties

The rheological properties of asphalt binder under intermedia and high temperatures were assessed with the dynamic shear rheometer (DSR) [29]. The temperature sweeps were conducted on the asphalt mortar at the frequency of 10 rad/s from 30 to 76 °C. The frequency sweeps were conduct on the asphalt mortar under 11 frequencies (1 rad/s, 1.58 rad/s, 2.51 rad/s, 3.98 rad/s, 6.31 rad/s, 10 rad/s, 15.8 rad/s, 25.1 rad/s, 39.8 rad/s, 63.1 rad/s, and 100 rad/s) at 60 °C.

The low temperature flexural creep stiffness of asphalt mortar was evaluated with the bending beam rheometer (BBR). Three different test temperatures (−18 °C, −12 °C, and −6 °C) were adopted.

#### 2.2.3. Asphalt Mixture Production

**Figure 1:**
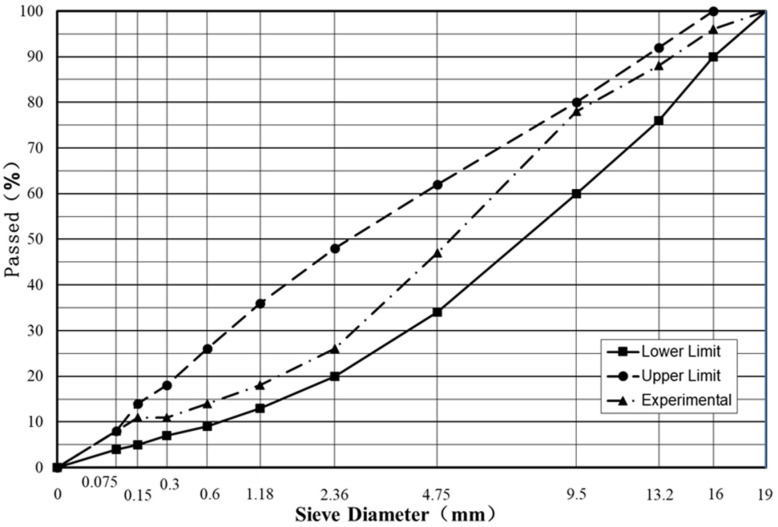
The gradation of the asphalt mixture.

The asphalt mixture was prepared with different fly ash contents (0%, 25%, 50%, 75%, and 100%) to replace the mineral filler. Based on previous research results that the optimum binder to aggregate ratio was not significantly influenced by the fly ash and mineral filler percentage, the binder to aggregate ratio was set as 4.5% for different types of asphalt mixture. The gradation of the aggregates is presented in Figure 1.

#### 2.2.4. Asphalt Mixture Properties

The high temperature property of the asphalt mixture was assessed with the Marshall stability test and the wheel tracking test. The Marshall stability measured the deformation resistance of the asphalt mixture under the cylindrical axis load with the Marshall apparatus. The test was conducted by following the ASTM D6927 standard [30]. The wheel tracking test generated the relationship between the rutting depth and the wheel tracking cycles. The samples were compacted by the rubber tire at 60 °C for 1 h at the speed of 42 passes/min [31]. Three replicate samples were conducted for each test condition. The dynamic stability was calculated with Equation (1).

(1) $$DS=\frac{42\times 15}{D_{60}-D_{45}}$$

where, *DS* is the dynamic stability (cycle/mm), *D*_60_ is the tracking depth at 60 min (mm), and *D*_45_ is the tracking depth at 45 min (mm).

The low temperature performance of the mixture was assessed with the indirect tensile strength test. The indirect tensile strength test was run at −10 °C, and the loading rate was 50 mm/min [32]. The test was operated according to the ASTM D6931 standard. Three replicate samples were conducted on each test condition.

The moisture stability characteristics of the asphalt mixture was assessed with the immersed Marshall test and the indirect tensile strength test after freeze-thaw cycles [33]. The Marshall stability of unconditioned sample (*MS_Control_*) and water bath conditioned sample (*MS_Conditioned_*) were evaluated, and the retained strength ratio (*RSR*) was calculated with Equation (2).

(2) $$RSR=\frac{MS_{Conditioned}}{MS_{Control}}\times 100$$

The indirect tensile strength test was operated by following the AASHTO T283. The conditioned samples were 70–80% saturated under the vacuum. The samples were then placed in a freezer at −18 °C for 16 h, after which the samples were thawed at 60 °C for 24 h [34]. The indirect tensile strength test of the samples under two conditions was compared to quantify the moisture sensitivity of the mixture.

## 3. Results and Discussions

### 3.1. Asphalt Mortar

#### 3.1.1. The Intermediate and High Temperatures Performance

**Figure 2:**
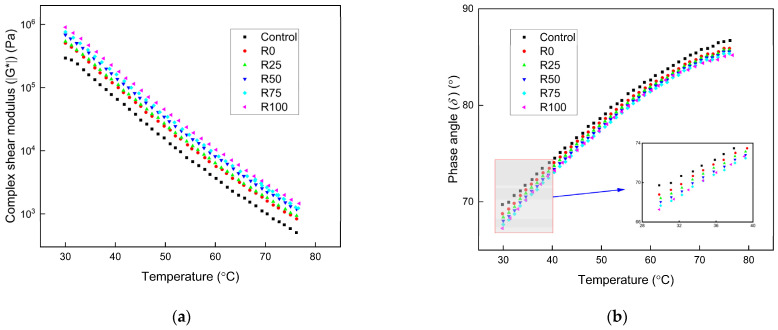
The temperature sweep results of asphalt mortar under the F/A of 0.6. (**a**) The complex shear modulus ǀG*ǀ under different conditions; (**b**) the phase angle (*δ*) under different conditions.

**Figure 3:**
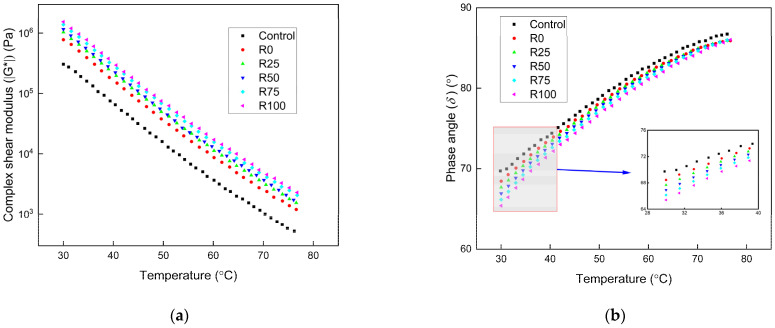
The temperature sweep results of asphalt mortar under the F/A of 0.8. (**a**) The complex shear modulus ǀG*ǀ under different conditions; (**b**) the phase angle (*δ*) under different conditions.

**Figure 4:**
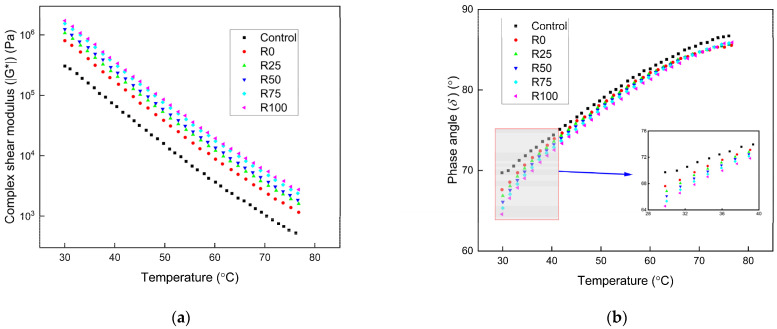
The temperature sweep results of asphalt mortar under the F/A of 1.0. (**a**) The complex shear modulus ǀG*ǀ under different conditions; (**b**) the phase angle (*δ*) under different conditions.

The temperature sweeps result of asphalt mortar under different test temperatures with the F/A ratios of 0.6, 0.8, and 1.0 are presented in Figure 2, Figure 3, and Figure 4, respectively. The complex shear modulus (ǀG*ǀ) decreased as the test temperature increased. The asphalt mortar stiffness decreased with the increase of the temperature since the asphalt mortar changed from elastic state to the viscous state, thus decreased the ǀG*ǀ. The phase angle (δ) increased with the increase of the test temperature, and the increasing speed was decreased at the high temperatures. The elastic components decreased, and the viscous components increased as the test temperature increased, thus increased the *δ*. The asphalt mortar was softened, the flowability increased at the high temperatures, and the temperature sensitivity of the *δ* was decreased.

At the same F/A ratio, the ǀG*ǀ of the asphalt mortar was significantly influenced by the addition of mineral filler and fly ash. The improvement was more significant for asphalt mortar with increased fly ash content. At the F/A ratio of 0.6, the ǀG*ǀ of asphalt mortar with fly ash was 80% higher than that with mineral filler. The improvement increased to 90–100% higher at the F/A value of 0.8. The value was 110–130% higher at the F/A value of 1.0. The addition of fly ash had more obvious improvement than mineral filler in the ǀG*ǀ of asphalt mortar. The high temperature performance of asphalt mortar with fly ash was better than that with mineral filler. Based on the fly ash and mineral filler properties in Table 3, the fly ash had higher surface area and lower water absorption rate. As a result of the higher surface area, more asphalt was absorbed by the fly ash, which enhanced the adhesion of asphalt. The interaction between the components of fly ash and asphalt binder also improved the adhesion and shear resistance of asphalt mortar. The ǀG*ǀ of asphalt mortar increased significantly as the F/A ratio changed from 0.6 to 0.8. The improvement was not apparent as the F/A ratio changed from 0.8 to 1.0. The high F/A ratio decreased the elasticity of asphalt mortar. The low F/A ratio weakened the shear resistance characteristics of asphalt mortar. The F/A ratio of 0.8 was the optimum value, which guaranteed the asphalt mortar had sufficient shear resistance and elasticity.

The *δ* of asphalt decreased as the fly ash and mineral filler was added. The impact of F/A ratio on the *δ* of asphalt mortar was reduced with the increase of F/A ratio. The impact of mineral filler on the *δ* of asphalt mortar was more significant than fly ash. The improved adhesion of asphalt mortar with fly ash increased the elastic components of asphalt mortar. The inclusion of fly ash and mineral filler did not remarkably influence the *δ* of asphalt mortar.

**Figure 5:**
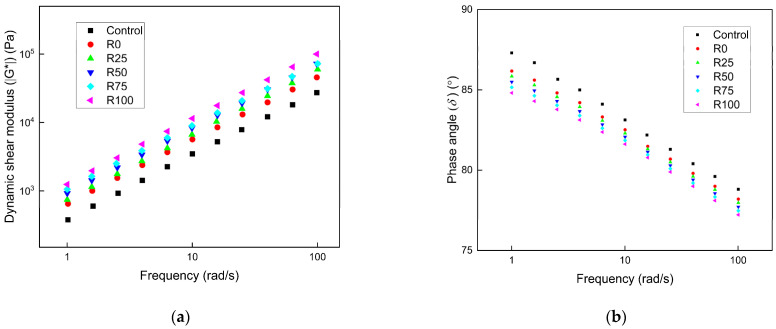
The frequency sweep results of asphalt mortar under the F/A of 0.6. (**a**) The complex shear modulus ǀG*ǀ under different conditions; (**b**) the phase angle (*δ*) under different conditions.

**Figure 6:**
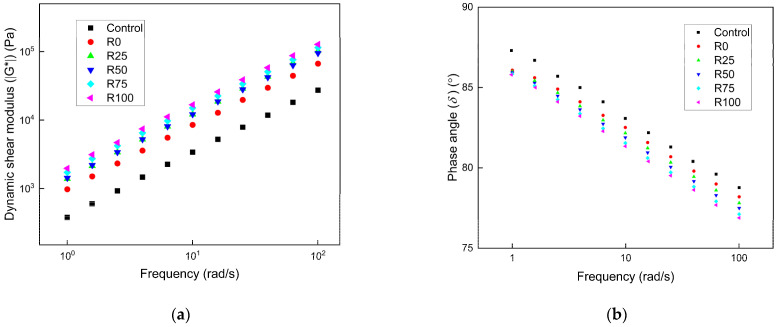
The frequency sweep results of asphalt mortar under the F/A of 0.8. (**a**) The complex shear modulus ǀG*ǀ under different conditions; (**b**) the phase angle (*δ*) under different conditions.

**Figure 7:**
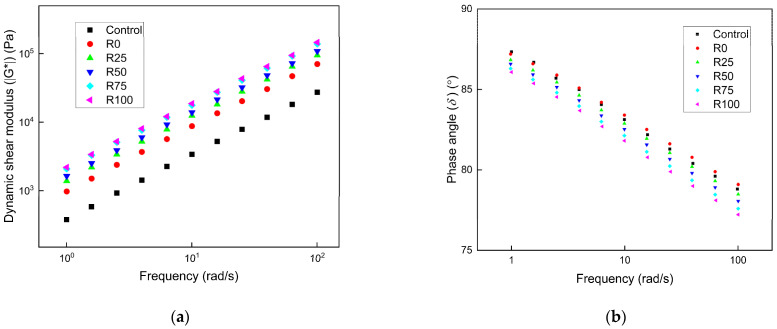
The frequency sweep results of asphalt mortar under the F/A of 1.0. (**a**) The complex shear modulus ǀG*ǀ under different conditions; (**b**) the phase angle (*δ*) under different conditions.

During the service period of the pavement, the pavement presented a different response to different types of traffic load frequencies. The pavement materials possessed different viscoelastic properties under different loading frequencies. The performance of asphalt mortar under different shear frequencies could represent the response of the asphalt pavement during different loading frequencies. The frequency sweep results of asphalt mortar under different F/A ratios are shown in Figure 5, Figure 6, and Figure 7, respectively. The ǀG*ǀ of asphalt mortar increased when the loading frequency increased, and the ǀG*ǀ had a linear relation with the frequency under the log scale. At the low frequency, the loading duration was longer, and the asphalt mortar was more prone to deform under the high temperature. The asphalt mortar had high sensitivity to the loading frequency. Under the same F/A ratio and loading frequency, the ǀG*ǀ of asphalt mortar with 100% fly ash (R100) was twice the value of asphalt mortar with 100% mineral filler (R0). The asphalt mortar with fly ash had higher shear resistance at high temperatures than asphalt mortar with mineral filler. The ǀG*ǀ of asphalt mortar was significantly increased as the F/A ratio changed from 0.6 to 0.8. The F/A ratio had limited improvement as the F/A ratio changed from 0.8 to 1.0. The *δ* of asphalt decreased as the frequency increased, and the *δ* of asphalt mortar with mineral filler was higher than that with fly ash. The *δ* of asphalt mortar was reduced when the F/A ratio increased, but the influence was minimal.

#### 3.1.2. The Low Temperatures Performance

**Figure 8:**
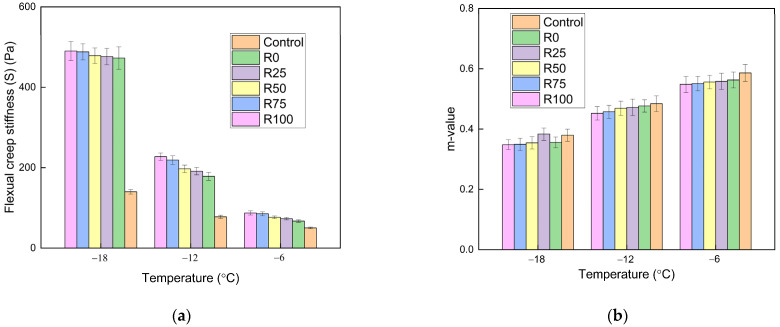
The BBR test results of asphalt mortar under the F/A of 0.6. (**a**) The stiffness under different conditions; (**b**) the m-value under different conditions.

**Figure 9:**
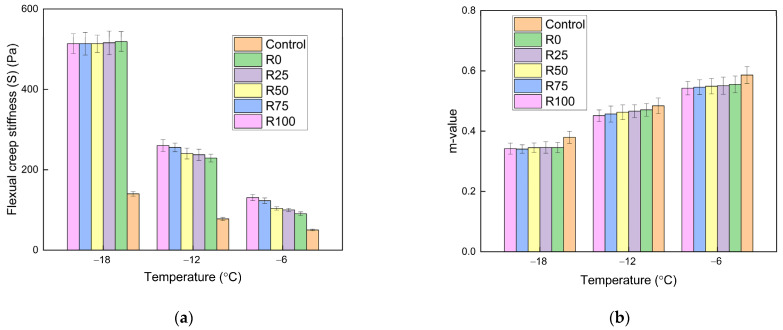
The BBR test results of asphalt mortar under the F/A of 0.8. (**a**) The stiffness under different conditions; (**b**) the m-value under different conditions.

**Figure 10:**
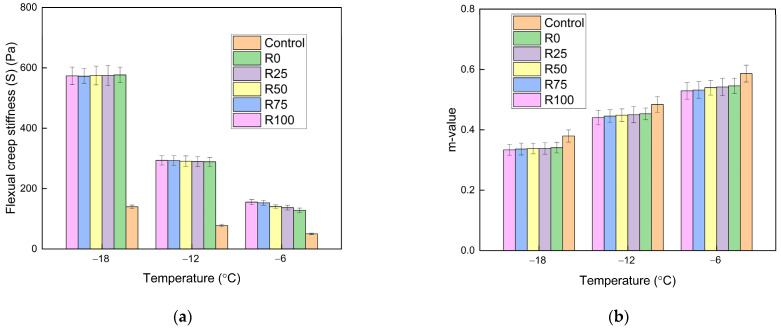
The BBR test results of asphalt mortar under the F/A of 1.0. (**a**) The stiffness under different conditions; (**b**) the m-value under different conditions.

At low temperatures, the asphalt mortar and asphalt mixture became hardened and brittle. The BBR test was adopted to identify the properties of asphalt mortar at low temperatures. The flexural creep stiffness (S) reflected the deformation resistance of the asphalt mortar. The m-value represented the relaxation of asphalt mortar. The cracking resistance of asphalt mortar could be characterized. The S and m-value of asphalt mortar with the F/A ratios of 0.6, 0.8, and 1.0 are shown in Figure 8, Figure 9, and Figure 10, respectively.

The asphalt without fly ash and mineral filler had the lowest stiffness and the highest m-value. After adding fly ash and mineral filler, the deformation resistance was decreased. The stiffness is enhanced and the m-value reduces as the temperature decreased. The temperature had a significant impact on the characteristics of asphalt mortar at low temperatures. At the same temperatures and F/A ratio, the stiffness and m-value linearly changed as the fly ash content increased. At a certain temperature, the stiffness was increased and the m-value decreased, when the F/A ratio increased. The stiffness decreased dramatically when the temperature decreased from −12 to −18 °C, which proved that the temperature sensitivity of stiffness was improved with the decrease of temperature. The differences between the stiffness of fly ash asphalt mortar and mineral filler asphalt mortar were reduced as the temperature decreased. At low temperatures, the filler type had minimal impact on the low temperature characteristics of asphalt mortar.

### 3.2. Asphalt Mixture

#### 3.2.1. The High Temperature Stability

The high temperature stability of the asphalt mixture reflected the permanent deformation resistance of the asphalt mixture under the impact of high temperature and traffic load [35]. The high temperature permanent deformation resistance was contributed to by the aggregate structure and the cohesive force of the asphalt binder. The physical effect and chemical reaction of the fillers influenced the shear strength, which provided lower contribution than the previous effects. The fly ash and mineral filler enhanced the high temperature property of the asphalt binder, thus influencing the permanent deformation resistance of the asphalt mixture. The high temperature property of the mixture was assessed with the Marshall stability and the wheel tracking test.

**Figure 11:**
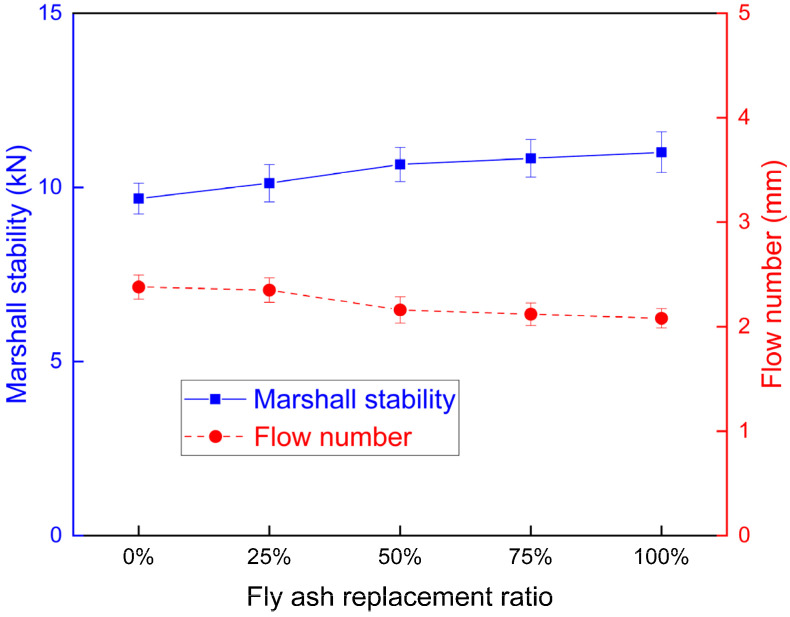
The Marshall stability and flow number of different mixtures.

The Marshall stability reflected the peak load during the test, and the Marshall flow defined the change of the mixture from the Marshall stability test. The Marshall stability and flow number of different asphalt mixtures are shown in Figure 11. The Marshall stability increased, and the flow number decreased, when the fly ash content increased, which enhanced the high temperature property of the asphalt mixture. The Marshall stability rate increase was higher when the ratio increased from 0 to 50%. Considering the burden to the environment of the asphalt mixture with too much fly ash during the production procedure, the fly ash replacement ratio was suggested to be less than 50%. The higher surface area of fly ash prompted a thicker asphalt film thickness, reduced the light component of asphalt in the mixture, thus increasing the high temperature performance of the mixture.

**Figure 12:**
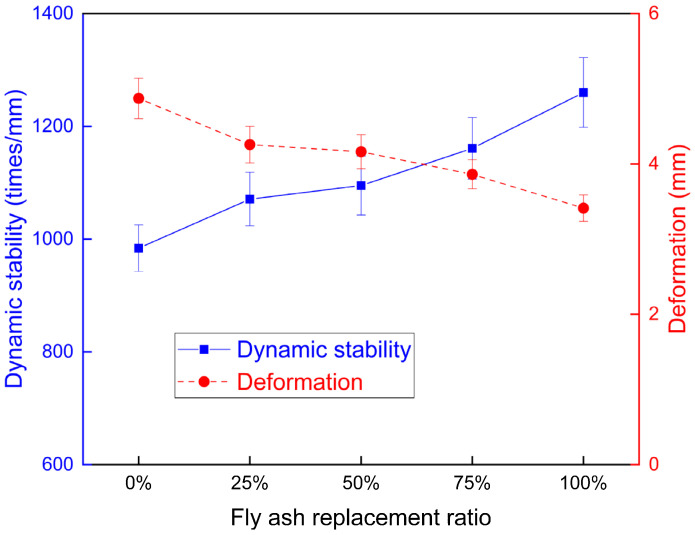
The dynamic stability and deformation of different mixtures.

The wheel tracking test simulated the rutting resistance of the mixture under high temperature and load [36]. The wheel tracking test was conducted on the asphalt mixture with different fly ash replacement ratios. The dynamic stability and total deformation were obtained from the wheel tracking test, as presented in Figure 12. The dynamic stability was increased, and the deformation of the asphalt mixture was reduced, when the fly ash percentage increased. The adoption of fly ash improved the high temperature property of the asphalt mixture, which is consistent with the Marshall stability results.

#### 3.2.2. The Low Temperature Cracking Property

**Figure 13:**
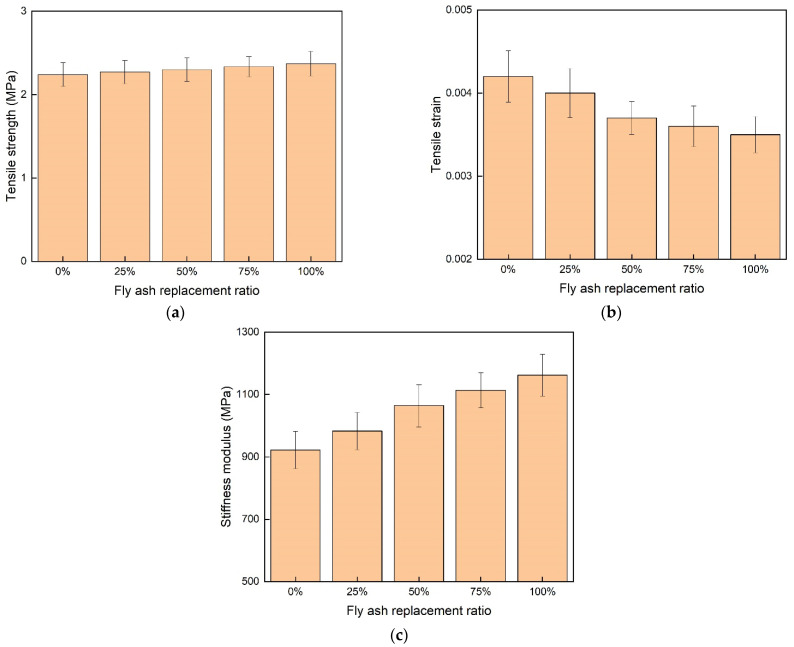
The low temperature cracking test results of asphalt mixture. (**a**) The tensile strength of different mixtures; (**b**) the tensile strain of different mixtures; (**c**) the stiffness modulus of different mixtures.

The asphalt mixture became hardened and brittle as the temperature decreased, the bonding failure between the asphalt binder and aggregate was the main cause for the asphalt mixture cracking at low temperatures. The cracking performance of the asphalt mixture was highly related to the performance of asphalt mortar. Based on previous property evaluation of asphalt mortar at low temperatures, the addition of fly ash slightly decreased the low temperature property. The low temperature indirect tensile strength test was adopted to quantify asphalt mixtures with different fly ash replacement ratios, as presented in Figure 13. The tensile strength of the asphalt mixture was linearly increased as the fly ash replacement ratio increased (Figure 13a). The addition of fly ash made the asphalt mixture stiffer and more hardened. The higher surface area of fly ash reduced the light components of the asphalt binder. The tensile strain of the asphalt mixture was decreased when the fly ash replacement ratio increased (Figure 13b). The elasticity of the asphalt mixture decreased as the hardness of the asphalt mixture was increased. The stiffness modulus of the asphalt mixture was increased as the fly ash replacement ratio increased (Figure 13b). One drawback of the hardened asphalt mixture was that the asphalt mixture became more brittle. Thus, the asphalt mixture was more susceptible to cracking at low temperatures. In order for the asphalt mixture to have sufficient cracking resistance at low temperatures, the fly ash replacement ratio should not be too high.

#### 3.2.3. The Moisture Susceptibility of the Asphalt Mixture

**Figure 14:**
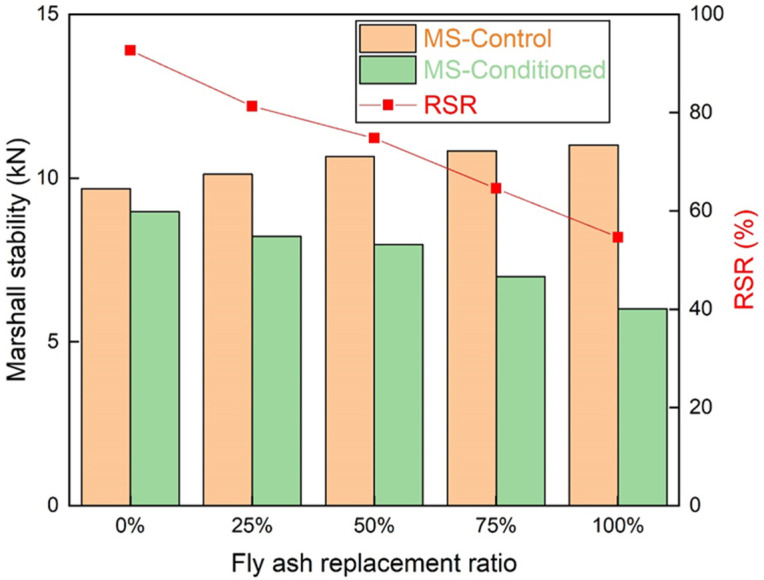
The immersed Marshall stability test results.

The moisture deterioration happened when the cohesion between the binder and aggregate was insufficient. The immersed Marshall test results are presented in Figure 14. The Marshall stability of unconditioned asphalt mixture improved as the fly ash replacement ratio increased. The Marshall stability of conditioned asphalt mixture reduced when the fly ash replacement ratio increased. Fly ash weakened the bonding of the asphalt mixture. The chemical components of fly ash may interact with water, and the intermolecular force was weakened. The bonding between the asphalt and aggregate was damaged, and stripping on the interface between asphalt and aggregate occurred. The *RSR* of the asphalt mixture reduced when the fly ash replacement ratio increased. Based on the standard, the *RSR* should be higher than 80%. The *RSR* decreased to lower than 80% when the fly ash replacement ratio was higher than 25%. In order to guarantee the moisture stability of the asphalt mixture, the fly ash replacement ratio should be set around 25%. If higher fly ash replacement ratio is demanded, an anti-stripping agent could be adopted, or the fly ash could be pretreated by washing with water to eliminate the influence of chemical components of fly ash on the asphalt mixture.

**Figure 15:**
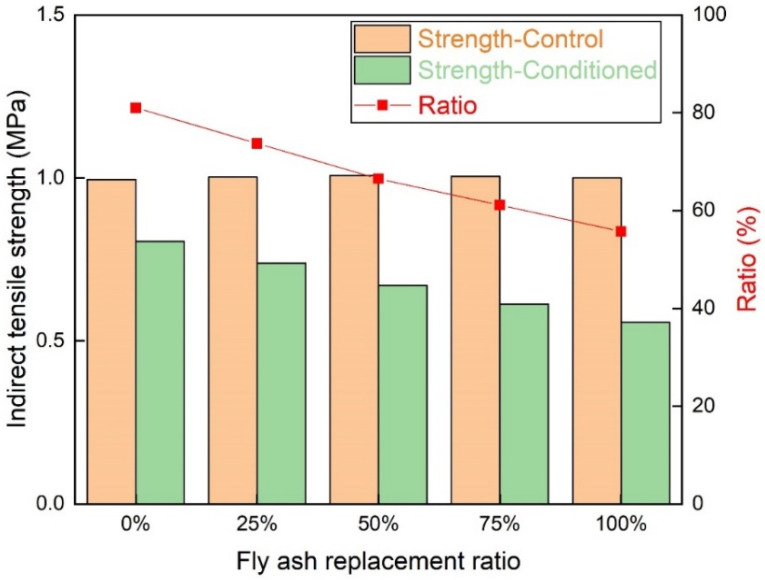
The indirect tensile strength of different mixtures.

The indirect tensile strength test condition was more severe than the immersed Marshall stability test. The samples experienced high percentage saturation, low temperature freeze, and high temperature thaw. The indirect tensile strength of different mixtures is presented in Figure 15. The indirect tensile strength of unconditioned samples increased slightly with the increase of the fly ash replacement ratio. After experiencing freeze-thaw cycles, the indirect tensile strength decreased significantly when the fly ash replacement ratio increased. The ratio of the conditioned sample to the unconditioned sample was reduced when fly ash replacement ratio increased. The decreasing trend was similar to that of the immersed Marshall stability test. All the samples with fly ash failed to meet the standard restriction. The fly ash replacement ratio of 25% was slightly lower than the standard value, due to the severe moisture condition. The indirect tensile strength test results were almost consistent with the immersed Marshall stability test results.

## 4. Conclusions

The impact of fly ash on the properties of asphalt mortar and asphalt mixture were evaluated. Mineral fillers were partially replaced with fly ash to produce asphalt mortar and asphalt mixture. The characteristics of asphalt mortar at different temperatures were assessed. The high temperature, low temperature, and the moisture susceptibility characteristics of the asphalt mixtures were assessed. The following conclusions can be obtained:

- The addition of fly ash enhanced the ǀG*ǀ and decreased the δ of the asphalt mortar. The high temperature performance of asphalt mortar with fly ash was better than asphalt mortar with mineral filler and base asphalt. The F/A ratio of 0.8 was the optimum value, which guaranteed that the asphalt mortar had sufficient elasticity and shear resistance.
- The asphalt without fly ash and mineral filler had the lowest stiffness and the highest m-value. After the addition of fly ash and mineral filler, the cracking resistance of asphalt at low temperatures was decreased. The filler type had insignificant impact on the characteristics of asphalt mortar at low temperatures.
- The Marshall stability and the wheel tracking tests’ results reached similar findings. The high temperature property of the mixture was enhanced with the increase of fly ash replacement ratio.
- The tensile strength and the stiffness modulus of the asphalt mixture were enhanced as the fly ash replacement ratio increased. The asphalt mixture with higher fly ash replacement ratio was more prone to low temperature cracking. The fly ash replacement ratio should not be too high, to ensure an asphalt mixture with sufficiently low temperature cracking resistance.
- The addition of fly ash weakened the moisture stability of the asphalt mixture, as predicted by the immersed Marshall test and the indirect tensile strength test results. In terms of sufficient moisture stability of the asphalt mixture, the fly ash replacement ratio should be set around 25%. If higher fly ash replacement ratio was demanded, the anti-stripping agent could be adopted, or the fly ash could be pretreated by washing with water to reduce the influence of chemical components of fly ash to the asphalt mixture. Even though the conclusions of the paper were based on limited mixture designs, the results could supply some experience for the mixture design with higher fly ash content and improved low temperature and moisture stability.

## Figures and Tables

**Table 1:** The performance of base asphalt.

Performance	Results
Softening point (°C)	47.1
Penetration (25 °C, 100 g, 5 s)/0.1 mm	71.7
Ductility (15 °C, 5 cm/min)/cm	>150
Flashing point (°C)	262
Viscosity (135 °C)/Pa∙s	0.435

**Table 2:** The basic performance of coarse aggregate.

Performance	Results
Crushing value (%)	20.7
Soundness (%)	4.0
Moisture content (%)	0.4
Water absorption (%)	0.5
Apparent specific gravity	2.736
LA abrasion loss (%)	16.7

**Table 3:** The properties of limestone mineral filler.

Properties		Test Results	
		Limestone Mineral Filler	MSWI Fly Ash
Apparent specific gravity		2.719	2.284
Moisture content (%)		0.4	0
Water absorption (%)		0.79	0.65
Specific surface area (m^2^/g)		1.39	6.48
Percentage passing	0.6 mm (#30)	100	100
	0.15 mm (#100)	97.8	95.7
	0.075 mm (#200)	78.3	77.5

**Table 4:** The element composition of MSWI fly ash.

Component	**O**	**Na**	**Mg**	**Al**	**Si**	**P**	**S**	**Cl**	**K**	**Ca**	**Ti**	**Cr**
Percentage (%)	26.254	2.029	0.569	0.56	1.752	0.163	2.569	22.265	3.397	37.888	0.168	0.022
Component	Mn	Fe	Ni	Cu	Zn	Br	Sr	Cd	Sn	Ba	Pb	
Percentage (%)	0.022	0.725	0.021	0.076	1.052	0.092	0.063	0.026	0.036	0.04	0.212	

**Table 5:** The dosage of fly ash and mineral filler in asphalt mortar.

Symbols	F/A Ratios	Fly Ash	Mineral Filler
R0	0.6, 0.8, and 1.0	0	100%
R25		25%	75%
R50		50%	50%
R75		75%	25%
R100		100%	0%

## Data Availability

The data that support the findings of this study are available on request from the corresponding authors.
